# Non‐Native Plants Attain Native Levels of Microherbivory Richness With Time and Range Expansion

**DOI:** 10.1111/ele.70247

**Published:** 2025-11-05

**Authors:** Lara J. Schulte, Miriam Wahl, Ingmar R. Staude

**Affiliations:** ^1^ Institute of Biology Leipzig University Leipzig Germany; ^2^ German Centre for Integrative Biodiversity Research (iDiv) Halle‐Jena‐Leipzig Leipzig Germany

## Abstract

Non‐native plants are often seen as peripheral to trophic networks due to a lack of co‐evolution with local biota, but the factors shaping their integration remain poorly understood. Using a continent‐wide dataset of 127,000 plant–microherbivore interactions across Europe, we show that native plants host more microherbivore species than non‐natives. Among non‐native plants, the number of associated microherbivore species was better predicted by time since introduction and range size in the introduced range than by relatedness to native flora or geographic origin. Species introduced more than two centuries ago, or with ranges as large as the average native plant, supported similar numbers of microherbivores as natives, though with a greater share of generalists. Our findings suggest that trophic networks can absorb novelty rapidly, with non‐natives attaining levels of microherbivory richness comparable to natives over relatively short timescales, but the persistence of specialised interactions remains dependent on native flora.

## Graphical abstract

Drawing on a continental database of over 120,000 plant–microherbivore interactions, we demonstrate that non‐native plants in Europe are not excluded from food webs indefinitely. With increasing residence time and range expansion, they can match native plants in microherbivory richness, although their interactions involve a greater share of generalist microherbivores.

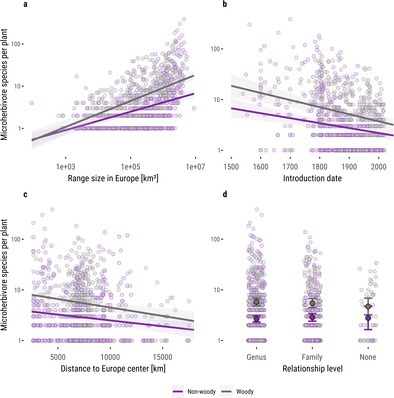

## 1 Introduction

Global change is reshaping plant biogeography, as more species establish beyond their historical ranges, forming novel combinations of species and interactions (Kerr et al. 2025; Ordonez et al. 2024). These non‐native populations are typically seen as threats to biodiversity (Lefebvre et al. 2024; Simberloff 2005). However, growing evidence shows that many of the species with non‐native populations are simultaneously declining in their native ranges, suggesting that extralimital populations may also offer opportunities for conservation (Lundgren et al. 2024; Staude et al. 2025). Whether non‐native populations should be eradicated or considered in conservation planning depends on their ecological fit in novel ecosystems, particularly their trophic interactions (Van der Putten et al. 2010). Microherbivores (small plant‐feeding organisms that live in or on living tissues, e.g., leaf miners, gall formers, borers, eriophyid mites and plant‐pathogenic fungi) are key components of plant‐associated trophic networks, and their absence in introduced ranges is thought to contribute to plant invasiveness, as proposed by the enemy release hypothesis (Keane and Crawley 2002; Mitchell and Power 2003). Understanding what drives interactions between microherbivores and non‐native plants is therefore crucial for insights into trophic integration and conservation planning.

Several mechanisms could explain why non‐native plants differ from native species in their trophic interactions with microherbivores (Meijer et al. 2016). Some of these mechanisms are rooted in long‐term evolutionary or biogeographic history and are fixed by the time a species is introduced (Brändle and Brandl 2001; Strong et al. 1984). Co‐evolution is typically invoked to explain reduced interactions in non‐native plants. Native species have evolved alongside local microherbivores over millennia, developing specialised relationships, whereas non‐native species typically lack such a history in their new range (Hill and Kotanen 2009; Pearse et al. 2013; Pearse and Hipp 2009). Still, non‐native plants with closely related congeners already present in the introduced region may be more likely to support microherbivores due to shared traits (Kirichenko et al. 2013; Pearse and Hipp 2009). Geographic proximity of the native range may also matter. That is, species arriving from nearby, ecologically similar areas might carry adaptations that facilitate trophic integration (Brändle and Brandl 2001; Kennedy and Southwood 1984). In some cases, pre‐existing interactions may even persist when the ranges of plants and their associated microherbivores overlap (Cripps et al. 2006). These historical and biogeographical factors represent constraints that are largely outside the influence of contemporary ecological processes.

But then there are also more dynamic factors unfolding over shorter ecological timescales. One key factor is a species' expansion in its introduced range (Brändle and Brandl 2001; Kennedy and Southwood 1984; Lawton and Schroder 1977). Widespread species are generally more likely to accumulate interactions simply because their larger ranges provide more opportunities for encounters with other organisms. For example, in Russia, conifers interact with more microherbivore species than deciduous trees, whereas in England, deciduous trees support more interactions than conifers; patterns that reflect the relative abundance of these plant groups in each region (Southwood 1961). This mirrors the species–area relationship observed in ecology (Connor and McCoy 1979). Instead of land area, here the ‘area’ is a plant's range size, and the total number of microherbivore species recorded per plant species scales accordingly (Lawton and Schroder 1977; Strong and Levin 1979). Another relevant factor may be the time since introduction. Non‐native species that have been present for centuries may have had more opportunities to accumulate interactions than more recent arrivals (Brändle et al. 2008; Kennedy and Southwood 1984). Unlike co‐evolutionary history or biogeographic origin, these factors may enable more dynamic patterns of trophic integration, unfolding not over millennia but over centuries.

Here, we use the largest plant–microherbivore interaction dataset for Europe to date, the Plant Parasites of Europe database (Ellis 2025), comprising 127,032 interactions between 12,183 plant species and 26,140 microherbivore species to examine the drivers of trophic integration (defined here as the richness of microherbivore species associated with each plant) in non‐native plants. We first tested whether non‐native plants interact with fewer microherbivores than native species. Next, we assessed whether microherbivory richness is shaped more by evolutionary legacy or by contemporary ecological processes. We examined four predictors: range size in the introduced range and time since introduction (reflecting dynamic, short‐term processes), and geographic proximity of the native range and relatedness to native flora (reflecting evolutionary or biogeographic legacy). Finally, we tested whether well‐integrated non‐native plants tend to interact with microherbivores that have broader host ranges, assessing the role of host specialisation in their integration. Our aim is to provide a macroecological perspective on the drivers of trophic integration and to evaluate whether non‐native plants remain ecological outsiders or gradually integrate into new ecosystems.

## 2 Methods

All data carpentry, visualisations and statistical analysis were performed in R (version 4.4.0) and are openly available on GitHub at: https://github.com/istaude/nonnatives‐microherbivores.

## 3 Data Synthesis

### 3.1 Microherbivore‐Plant Interactions

We used the Plant Parasites of Europe database, the most comprehensive dataset on plant–microherbivore interactions in Europe to date (available at https://bladmineerders.nl; Ellis 2025). The dataset was compiled by Willem Ellis over the past 25 years and generously provided in CSV format for this study. It contains documented interactions between 26,140 microherbivore species and 12,183 plant taxa, totaling 127,032 interactions. The dataset does not include free‐feeding folivores (e.g., caterpillars). Instead, it is dominated by internal or tissue‐modifying groups: leaf miners, gall formers, borers and eriophyid mites (‘vagrants’), and by plant‐pathogenic fungi (leaf spots, pustules, rusts, powdery/downy mildews). It also includes some sap‐feeding Hemiptera (scale insects). Many of these groups tend to exhibit higher host specificity than free‐feeding herbivores (Forister et al. 2015). For an overview of the frequency of each guild, see Figure S1. The dataset spans all of Europe, including European Russia and Turkey, and is based on 14,898 published sources since 2001. The dataset does not distinguish between native and non‐native microherbivores. Native range maps are rarely available and, where they exist, are often outdated or incomplete, making a systematic classification infeasible (W. Ellis, pers. comm.). While some microherbivores have established or been co‐introduced with their hosts, these are rare exceptions compared to the thousands of native taxa (Santini et al. 2013). We therefore expect non‐native species to represent only a small fraction of the 26,140 microherbivores in our dataset. We harmonised plant species names to the taxonomy of the World Checklist of Vascular Plants (WCVP; Govaerts et al. 2021) using the rWCVP package (Brown et al. 2023). After harmonising species names and excluding records assigned only to plant genera, the dataset included documented interactions for 10,474 accepted plant species. For each accepted plant species, we calculated the number of associated microherbivore species. This number reflects *documented* plant–microherbivore associations from the literature, acknowledging that, as with any such synthesis, some associations remain unknown.

### 3.2 Non‐Native Plant Species

To determine which plant species are native to Europe, we used distribution data from the WCVP, which includes 1,970,252 plant locality records globally. We first retrieved all botanical country codes corresponding to Europe using the *get_wgsrpd3_codes()* function from the rWCVP package (Brown et al. 2023; Figure S2). Botanical countries are standardised geographical units defined by the World Geographical Scheme for Recording Plant Distributions (WGSRPD), designed to be more comparable in size than political countries. For each taxon–region combination, the WCVP indicates its status as either native or introduced. We classified a species as native to Europe if it was listed as native in at least one European botanical country. We joined plant origin information to the species in our interaction dataset, excluding those lacking distribution records. In total, 10,395 plant species had both interaction and distribution data available, comprising 6860 native and 3535 non‐native species.

### 3.3 Plant Woodiness

Because woodiness is a strong predictor of microherbivore interactions (Kozlov et al. 2015; Turcotte et al. 2014; Wallace and Mansell 1976), differences between native and non‐native species could be confounded by this trait. To better isolate the effect of plant origin, we included woodiness in our analyses. We used a comprehensive database covering life cycle and woodiness traits for 235,000 plant species (Poppenwimer et al. 2023). We harmonized species names in this trait dataset using the rWCVP package (Brown et al. 2023) to align with the taxonomy used in our interaction dataset. After joining the datasets, woodiness information was available for 78% (*n* = 7874) of plant species. For the remaining species, we used a phylogenetic gap‐filling approach (Swenson 2014), wherein woodiness was inferred by assigning the modal (most frequent) woodiness state within the genus to species lacking data. This approach yielded woodiness information for 10,265 species (130 remained unclassified due to lack of data within their genus).

### 3.4 Range Size of Non‐Native Plants in Europe

Next, we compiled macroecological data that could influence the number of microherbivore associations on non‐native plants. As a first step, we estimated each non‐native species' area of occupancy (AOO) in Europe using occurrence records from the Global Biodiversity Information Facility (GBIF), accessed via the rgbif package (Chamberlain et al. 2024). For each species, we retrieved a GBIF usageKey using the *name_backbone()* function, which interfaces with GBIF's taxonomic backbone; any missing keys were added manually. To restrict occurrences to Europe, we prepared a spatial shapefile of European botanical regions. We used the *get_wgsrpd3_codes()* function from the rWCVP package (Brown et al. 2023) to retrieve WGSRPD botanical country codes, which we matched to WGSRPD shapefiles (https://github.com/tdwg/wgsrpd) and filtered to include only European regions. We reprojected the shapefiles to an equal‐area, Europe‐centered coordinate reference system (CRS; EPSG:8857) using *st_transform()* from the sf package (Pebesma 2018).

We used the GBIF usageKeys with *mvt_fetch()* from the rgbif package (Chamberlain et al. 2024) to retrieve map vector tiles for each species. The function was parameterized to: (1) include only records with the basis of record ‘human observation’, ‘machine observation’, or ‘observation’ (i.e., fossil specimens, living specimens from zoos and botanical gardens, and preserved specimens from museums and herbaria were excluded); (2) exclude records with coordinate issues; and (3) aggregate occurrences at ‘squareSize = 8’, corresponding to ~2500 km^2^ resolution in Central Europe, using the default Web Mercator projection (EPSG:3857). To match the European shapefile, results were reprojected to the equal‐area CRS EPSG:8857. We used the sf package (Pebesma 2018) to spatially crop species distributions to Europe using the *st_intersection()* function, and calculated non‐native AOOs using *st_area()*. This process yielded AOO estimates for 1902 out of 3535 non‐native plant species (53.8%). While AOO estimates are subject to sampling biases, the relatively high sampling density in Europe is likely to make them a reasonable, if coarse, proxy for non‐native range size (Staude et al. 2022).

### 3.5 Non‐Native Plant Introduction Dates in Europe

We obtained earliest introduction dates for non‐native plants in Europe from the Alien Species First Record database (Seebens et al. 2018; https://zenodo.org/records/3690742), which includes 77,375 first records of alien species globally, 17,611 of which refer to vascular plants in Europe. Because the database contains separate entries for different European regions, we selected the earliest record per species in Europe. After taxonomic harmonisation with WCVP, we merged these data with our interaction dataset, yielding 1005 non‐native species with introduction dates. Of these, 998 species (99%) were introduced after the year 1492, a common threshold for defining non‐native species because it marks the onset of large‐scale species exchanges during the Columbian Exchange. The remaining seven included two likely errors (e.g., *Nicotiana rustica* , *Phytolacca americana* ) and five species with genuine pre‐1492 records (e.g., *Cannabis sativa* , *Ficus elastica* , 550 BCE–1492 CE). Because these records are outside our operational definition and would introduce a large gap in the distribution of introduction times that may inflate leverage, we excluded them. In total, we retained 998 non‐native species with introduction dates.

### 3.6 Native Range Centroids of Non‐Native Plants

To calculate the geographic distance between each species' native range centroid and the center of Europe, we used WCVP distribution data accessed through the rWCVP package (Brown et al. 2023). We first determined the geographic center of Europe by merging all European botanical countries (Figure S2; see above) into a single shapefile using the *st_union()* function from the sf package (Pebesma 2018), and then computing the centroid with *st_centroid()*. This yielded a mean centroid of Europe at 59.56°N, 28.21°E. Next, we calculated the native range centroid for each non‐native species. Using the *wcvp_distribution()* function, we retrieved distribution data and filtered it to retain only native botanical countries. We then calculated the centroid of each botanical country and averaged these to obtain a single native range centroid per species. Finally, we used the *distGeo()* function from the geosphere package (Hijmans et al. 2024) to compute the great‐circle distance (in kilometres) between each species' native range centroid and the European centroid. We obtained geographic distance values for 3533 non‐native plant species. To avoid artificial proximity caused by averaging disjunct ranges (e.g., species native to multiple continents), we excluded species whose native range centroid fell within 2500 km of Europe's center (approximately half the continent's diameter), removing 17 species.

### 3.7 Relatedness to Native Plants

To assess the phylogenetic relatedness of non‐native plants to the native European flora, we classified each non‐native species into one of three categories: (1) congeneric (sharing a genus with at least one native species), (2) confamilial (sharing a family but not a genus), or (3) unrelated (no genus or family shared with any native species). Using WCVP distribution data accessed via rWCVP (Brown et al. 2023), we identified all genera and families native to Europe: 1572 genera and 157 families according to WCVP. We then matched non‐native plant species from our plant‐microherbivore interaction dataset by comparing their genus and family to those of the native European flora. Of the 3535 non‐native plant species, 2484 (70.3%) had native congeners, 911 (25.8%) were confamilial, and 140 (4.0%) had no relatives in Europe.

## 4 Data Analysis

### 4.1 Comparing the Number of Associated Microherbivore Species Between Native and Non‐Native Plants

To test whether microherbivore richness differed between native and non‐native plants, we fitted a linear mixed‐effects model with log_10_‐transformed microherbivore richness as the response variable. The transformation was applied to account for the heavy‐tailed distribution, characterised by many plants interacting with a single microherbivore species and only a few interacting with many. Fixed effects included plant origin, woodiness, and their interaction. To account for phylogenetic non‐independence, we included random intercepts for plant family. The model was fitted using the lme4 package (Bates et al. 2014), and *p*‐values were obtained using the lmerTest package (Kuznetsova et al. 2017). Model assumptions were assessed with the DHARMa package(Hartig et al. 2024), which indicated some deviation from normality in the residuals. To verify the robustness of our results, we refitted the model using a robust linear mixed‐effects approach implemented in the robustlmm package (Koller 2016). *P*‐values were calculated with the *tab_model()* function from the sjPlot package (Lüdecke et al. 2025). We analysed 5751 native non‐woody, 2074 non‐native non‐woody, 1061 native woody, and 1379 non‐native woody plant species.

### 4.2 Explaining Variation in Microherbivore Richness on Non‐Native Plants

To identify drivers of microherbivore richness on non‐native plants, we fitted a single model including all key predictors (i.e., range size, introduction date, geographic proximity and relatedness) to account for potential confounding. For instance, introduction date and range size were correlated (Figure S3), likely because earlier introductions had more time to expand. A total of 942 non‐native plant species had complete data for all variables. We fitted a linear mixed‐effects model with the number of associated microherbivore species (log_10_‐transformed) as the response variable. Fixed effects included log_10_‐transformed range size (km^2^), earliest recorded introduction date (year), geographic proximity of the native range (km), relatedness to native plant species (categorised as genus, family, or none), woodiness, and the interaction between woodiness and each of the other predictors. To account for phylogenetic non‐independence, plant family was included as a random intercept. The model was fitted using the lme4 package (Bates et al. 2014), and *p*‐values were obtained using the lmerTest package (Kuznetsova et al. 2017). Model assumptions were assessed with the DHARMa package (Hartig et al. 2024), and no major violations were detected. To estimate slopes and standard errors separately for woody and non‐woody species, we used the emmeans package (Lenth et al. 2025). Model explanatory power was evaluated by calculating marginal and conditional *R* ^2^ using the MuMIn package (Bartoń 2025). We quantified the contribution of each predictor to the explained variance with the partR2 package (Stoffel et al. 2021).

### 4.3 Host Breadth of Microherbivores on Trophically Well‐Integrated Non‐Native Plants

We tested whether non‐native plant species that, according to the previous analysis, support native‐like levels of microherbivore richness tend to interact with more generalist microherbivores than native plants. For each microherbivore, host breadth was the number of plant species (native and non‐native) used in the full dataset (Forister et al. 2015). For each plant species, we averaged the host breadths of its interacting microherbivores to obtain a per‐plant host‐breadth metric; no specialist–generalist threshold was imposed, and larger values indicate greater generalism. We modelled host breadth with a linear mixed‐effects model including plant origin, woodiness, and their interaction as fixed effects, and plant family as a random intercept to account for phylogenetic non‐independence. Non‐native plants were limited to those predicted to interact with native‐level microherbivory richness in the previous analysis, with growth‐form‐specific range‐size thresholds (> 270,000 km^2^ woody; > 1.1 million km^2^ non‐woody) reflecting the steeper richness–range relationship in woody species (Figure 2a). We analysed 5751 native and 156 non‐native non‐woody species, and 1061 native and 201 non‐native woody species.

To provide additional insight into the specialisation of interacting microherbivores, we derived a categorical host‐specialisation index following Bassi and Staude 2024. For each microherbivore species, we counted the number of distinct host plant species, genera, and families recorded in the dataset and assigned one of four categories: monophagous (one host species), oligophagous (> 1 host species across ≤ 4 genera within one family), mesophagous (> 1 host species across > 1 genus within ≤ 3 families), or polyphagous (> 1 host species across > 1 genus within > 3 families). For each plant species, we then calculated the composition of its interaction partners as the proportion of associated microherbivores in each category. We summarised these per‐plant proportions by averaging across plants within plant origin × woodiness groups to show the composition of microherbivore specialisations by plant origin and woodiness. Finally, we ran an exploratory supplementary analysis testing whether microherbivore specialisation composition shifts with residence time among non‐native plants. Among the set of trophically well‐integrated non‐native plants with introduction times available (*n* = 307), we binned residence time and, for each bin, displayed the weighted mean per‐plant composition of microherbivore specialisations (mono/oligo/meso/poly), with weights equal to each plant's total recorded interactions.

## 5 Results

### 5.1 Microherbivores and Plant Origin

**FIGURE 1:**
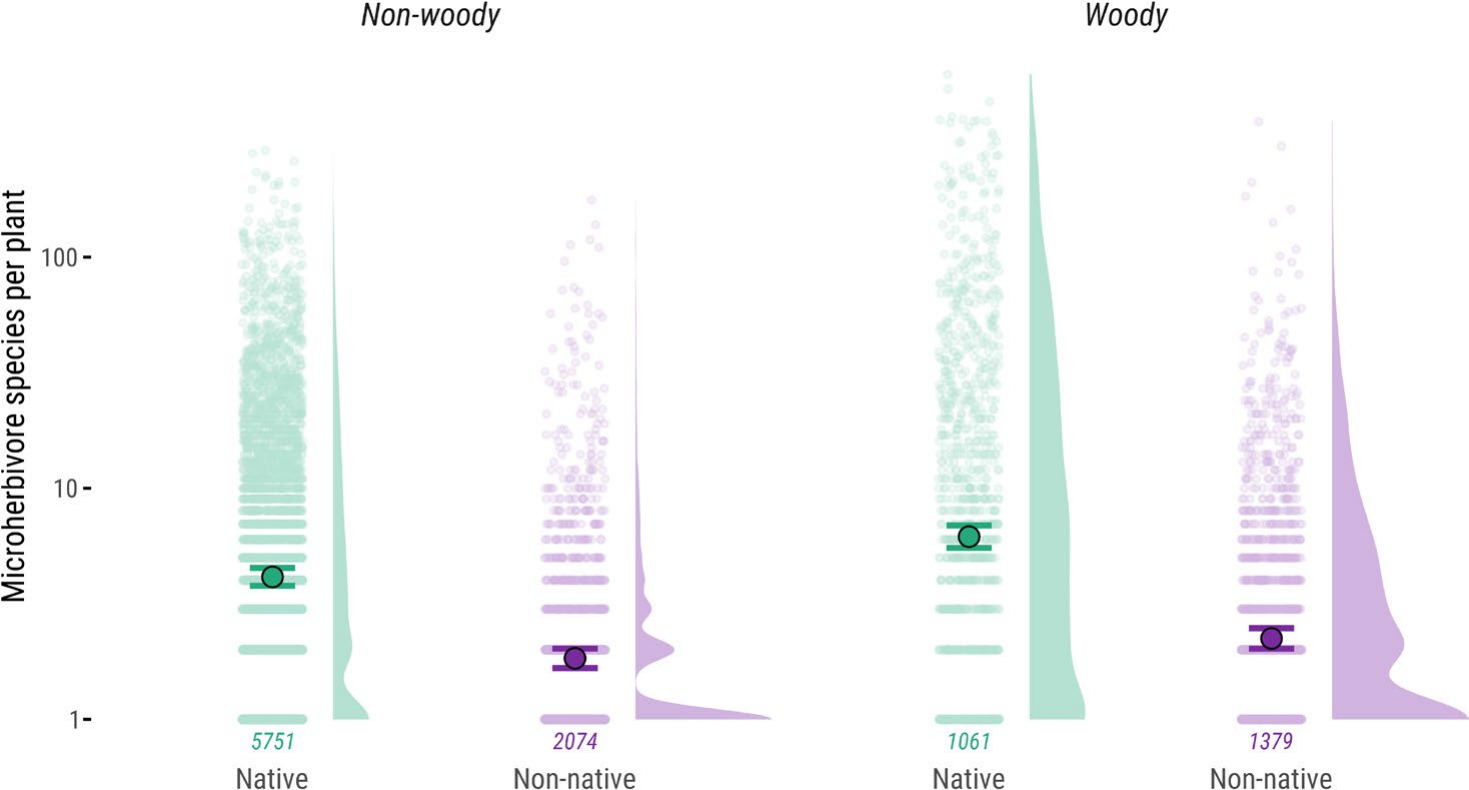
Native plants support, on average, a higher number of associated microherbivore species than non‐native plants. Plants are grouped by woodiness (non‐woody vs. woody) and by origin (native = green, non‐native = purple). Each point represents one plant species (semi‐transparent), with coloured points indicating group means and error bars showing 95% confidence intervals. Density plots show the distribution of values. The y‐axis is on a log_10_ scale. Sample sizes are indicated below the point clouds.

Non‐native plants supported fewer microherbivore species than native plants. Native woody plants hosted 6.17 ± 0.35 microherbivore species (mean ± standard error), compared with 2.24 ± 0.12 for non‐native woody plants. Similarly, native non‐woody plants supported 4.13 ± 0.19 microherbivore species, whereas non‐native ones hosted 1.84 ± 0.09. This represents a 2.75‐fold increase for woody plants and a 2.24‐fold increase for non‐woody plants in microherbivore richness on natives (Figure 1). While woodiness also affected microherbivore richness, its effect was smaller in magnitude. Within native plants, woody species supported 1.49 times more microherbivores than non‐woody species; within non‐native plants, the fold increase was 1.22. Plant origin (F(1, 10,027) = 926.6, *p* < 0.001), woodiness (F(1, 4943) = 61.37, *p* < 0.001), and their interaction (F(1, 10,122) = 11.73, *p* < 0.001) were all statistically significant predictors. Results remained consistent when we refitted the model using robust estimation to reduce the influence of the heavy‐tailed response distribution (Table S1).

### 5.2 Drivers of Trophic Integration of Non‐Native Plants

To understand the drivers of trophic integration in non‐native plants, we modeled multiple ecological and evolutionary predictors simultaneously. Specifically, we tested how range size, residence time, native‐range proximity to central Europe, and relatedness to native flora (presence of a congener or confamilial), each interacting with woodiness, influences the number of associated microherbivores. This approach controls for confounding relationships, such as the tendency for earlier‐introduced species to have larger European ranges (Figure S3). Reported effects thus reflect marginal contributions, having adjusted for all other variables in the model. Together, the fixed effects explained 36.3% of the variance in microherbivore richness across non‐native plants (marginal *R* ^2^), increasing to 42.6% when accounting for phylogenetic structure via random intercepts for plant family (conditional *R* ^2^).

#### 5.2.1 The Effect of Range Size

Non‐native plant species that were more widespread in Europe interacted with more microherbivore species (Figure 3a). In non‐woody species, a tenfold increase in European range size corresponded to a 1.65‐fold increase in the number of associated microherbivore species (slope = 0.218 ± 0.023 SE, *p* < 0.001; log_10_–log_10_ scale). In woody species, the effect was even stronger, with richness doubling for every tenfold increase in area (2.03‐fold increase; slope = 0.308 ± 0.031 SE, *p* < 0.001). Non‐native species with a European range size of ≈1.1 million km^2^ (non‐woody) and ≈270,000 km^2^ (woody) hosted as many microherbivore species as the average native plant (4.13 for non‐woody and 6.14 for woody native species). As a reference, our analysis estimated the average range size of native plants in Europe to be ≈1,000,000 km^2^.

#### 5.2.2 The Effect of Introduction Date

Non‐native plant species introduced earlier to Europe supported more microherbivore species (Figure 3b). In non‐woody species, a 100‐year earlier introduction was associated with a 25% increase in the number of associated microherbivores (slope = −0.00097 ± 0.00023 SE, *p* < 0.001; response on log_10_ scale). In woody species, the effect was slightly stronger, with a 100‐year earlier introduction corresponding to a 38% increase in richness (slope = −0.0014 ± 0.00027 SE, *p* < 0.001). Non‐native species introduced around the year 1719 (non‐woody) and 1845 (woody) supported as many microherbivore species as the average native plant.

#### 5.2.3 The Effect of Native‐Range Geographic Proximity

Non‐native plant species with native ranges geographically closer to Central Europe hosted more microherbivore species (Figure 3c and Figure S4). Across the observed range of distances in our dataset (2654–17,523 km), species with the closest native‐range centroids hosted 46% (non‐woody) and 67% (woody) more microherbivore species than those from the most distant ones (slopes = −2.31 × 10^−5^ ± 7.75 × 10^−6^ SE, *p* < 0.001, and −3.33 × 10^−5^ ± 9.42 × 10^−6^ SE, *p* < 0.001, respectively; response on log_10_ scale; percent increases were calculated as exp(β × Δdistance), where Δdistance = −14,868 km).

#### 5.2.4 The Effect of Relatedness to Native Plants

Relatedness to the native flora did not significantly influence microherbivore richness in non‐native species (Figure 3d). Among non‐woody plants, estimated richness was similar whether a species shared a genus with the native flora (≈2.7 species), only a family (≈2.9), or had no relatives (≈2.8), with all pairwise comparisons non‐significant (*p* > 0.69). Woody plants showed a comparable pattern, with estimated richness of ≈5.8, 5.5 and 4.7 species, respectively (*p* > 0.57).

#### 5.2.5 Variance Partitioning

Variance partitioning revealed that range size (i.e., European area of occupancy, together with its interaction with woodiness) explained the largest share of variation in microherbivore richness among non‐native species (semi‐partial *R* ^2^ = 11.0%, 95% CI: 6.1%–17.8%). In contrast, time since introduction and geographic proximity (each including their interaction with woodiness) accounted for more modest proportions (3.5% and 2.1%, respectively), while relatedness to the native flora explained very little unique variation (0.4%). Note that semi‐partial *R* ^2^ values reflect only the variance uniquely attributable to each predictor and do not sum to the full marginal *R* ^2^ (36.3%), which also includes variance jointly explained by multiple predictors.

**FIGURE 2:**
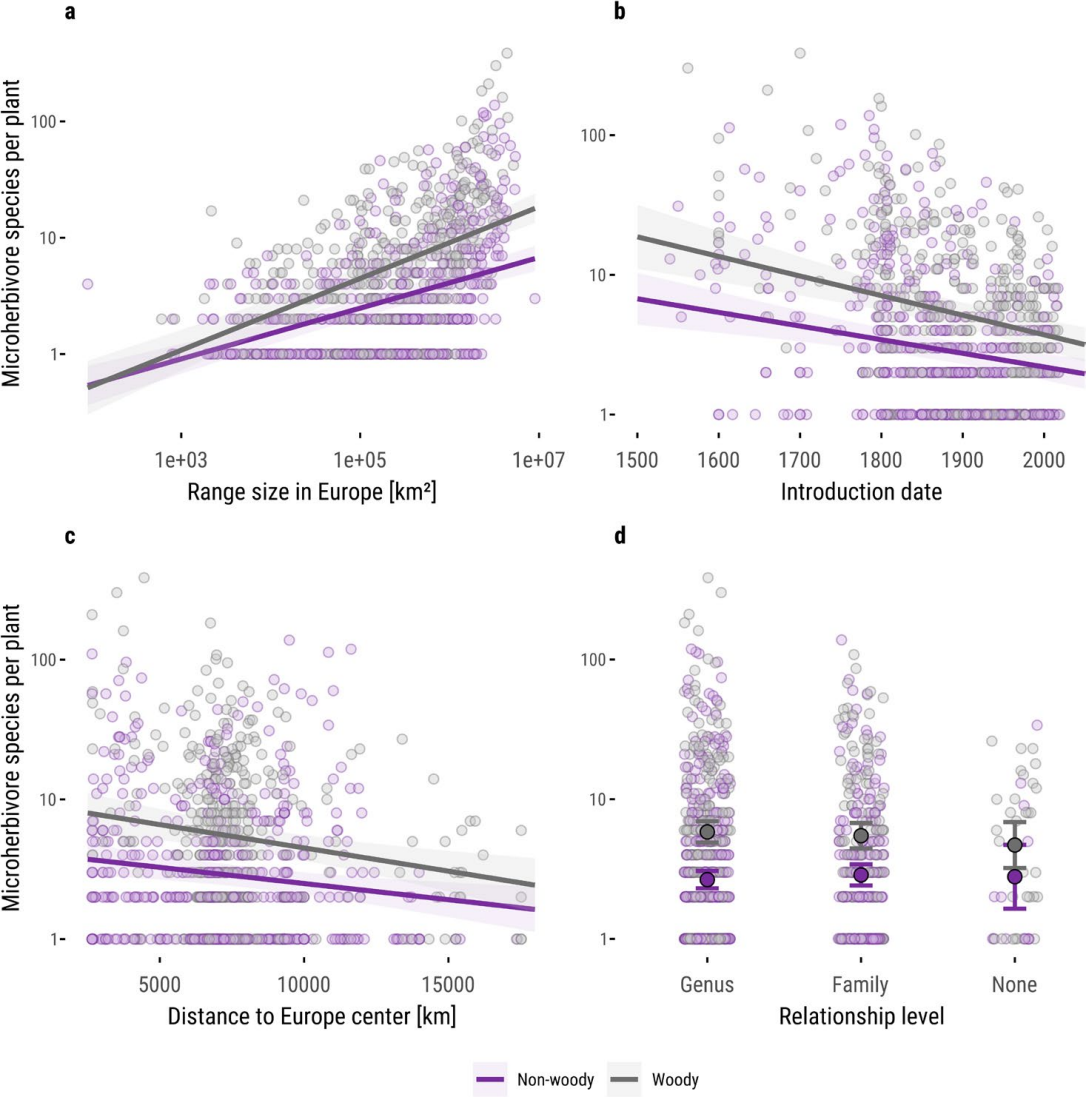
Drivers of the number of microherbivore species associated with non‐native plant species in Europe. (a) Microherbivory richness increases with non‐native plant species' area of occupancy (range size) in Europe, with a steeper slope for woody than non‐woody plants. (b) Earlier introduction dates are associated with higher microherbivory richness. (c) Microherbivory richness declines with increasing geographic distance between a non‐native plant species' native range centroid and the center of Europe. (d) Microherbivory richness does not differ significantly depending on the presence of a congener, confamilial, or no close relative in the native European flora. Semi‐transparent points show observed values (log_10_ scale on y‐axis) for each non‐native plant species (*n* = 942); lines and shaded bands, as well as coloured points and error bars, represent marginal effects with 95% confidence intervals, predicted from a linear mixed‐effects model including all predictors simultaneously. Non‐woody species are shown in purple, woody species in grey.

### 5.3 Specialisation of Microherbivores and Non‐Native Plants

**FIGURE 3:**
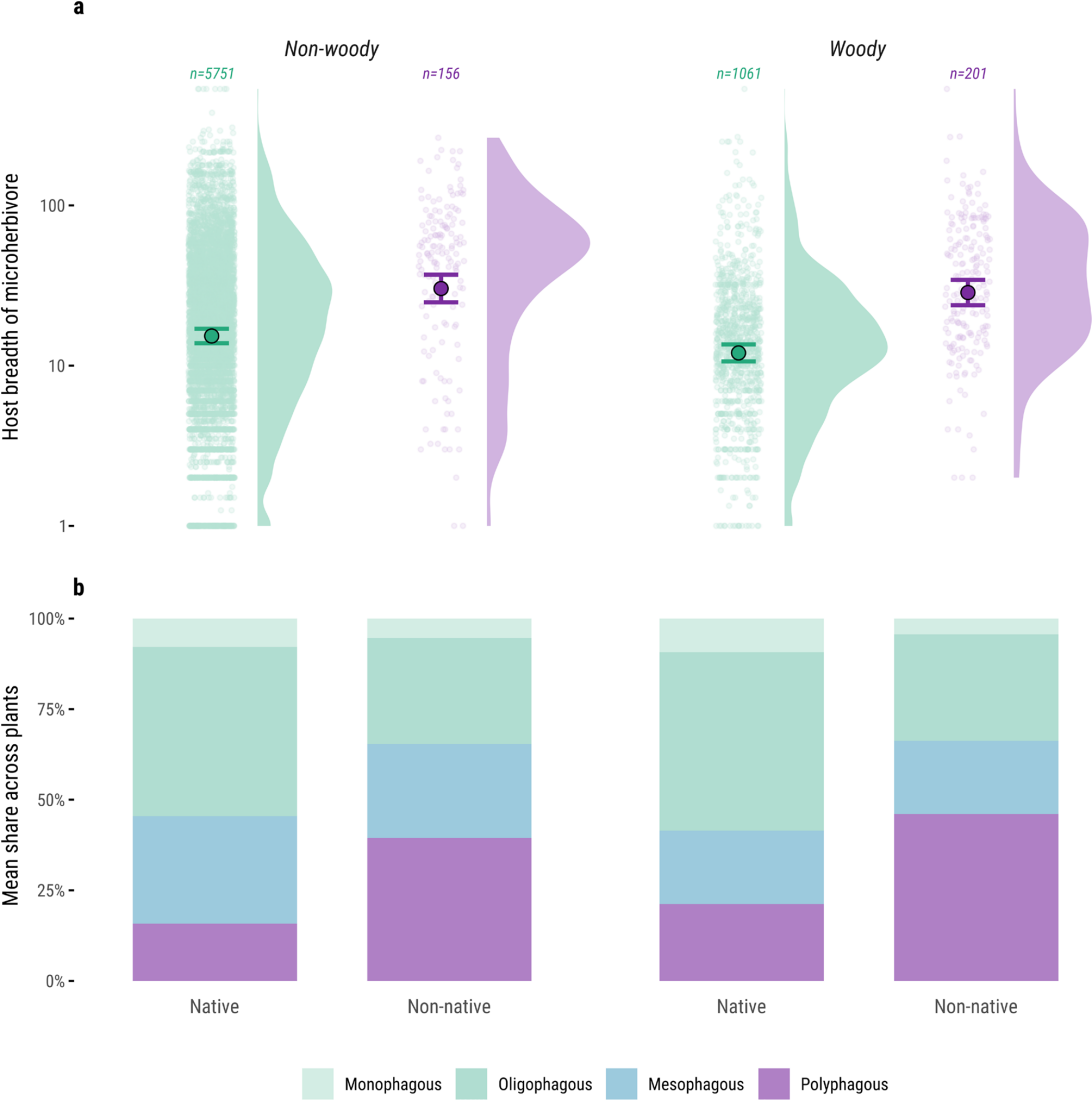
Trophically well‐integrated non‐native plants tend to interact with more generalist microherbivores than native plants. (a) Per‐plant mean host breadth of associated microherbivores (where host breadth is the number of plant species used; log_10_ scale). Semi‐transparent points represent plant species; coloured points and error bars are estimated marginal means ±95% CI. Non‐native plants shown are those exceeding the trophic‐integration range thresholds (> 1.1 million km^2^ for non‐woody, > 270,000 km^2^ for woody species; cf. Figure 2). Sample sizes are printed above the point clouds. (b) Average per‐plant specialization of microherbivores classified as mono‐, oligo‐, meso‐, or polyphagous. Bars show the mean proportion of each specialization class per plant species, averaged within plant origin × woodiness groups (e.g., the average non‐woody native plant interacts with 8% mono, 27% oligo, 30% meso and 15% polyphagous microherbivores).

Next, we asked whether non‐native plants, despite eventually supporting similar numbers of microherbivore species, tend to interact with more generalist microherbivores than native plants. If so, they may fail to substitute for the many specialized interactions maintained by natives (Figure S5). We calculated each microherbivore's host breadth (the number of plant species it feeds on) and focused on non‐native plants with large European range sizes (≈1.1 million km^2^ for non‐woody and ≈270,000 km^2^ for woody species), which were previously shown to best predict native levels of microherbivory richness (Figure 2 and Figure S6). We found that microherbivores associated with non‐native plants had significantly broader host ranges than those associated with native plants (Figure 3a), averaging 30.3 vs. 15.3 plant species for non‐woody species and 28.6 vs. 12.0 for woody ones. Plant origin had a strong effect on host breadth (F(1, 6751) = 147.89, *p* < 0.001), woodiness had a smaller but significant effect (F(1, 4994) = 4.64, *p* = 0.031), and their interaction was not significant (F(1, 6902) = 2.17, *p* = 0.14). Results were consistent when we classified microherbivores as mono‐, oligo‐, meso‐, or polyphagous (Figure 3b). Non‐native plants had higher shares of polyphagous and lower shares of mono‐ and oligophagous microherbivores than natives, in both woody and non‐woody plants. We found no clear shift in specialization composition of microherbivores on non‐native plants with increasing residence time (Figure S7).

## 6 Discussion

Here, we used a novel synthesis dataset on plant–microherbivore interactions in Europe, to test whether and why non‐native plant species differ from native species in the number of associated microherbivore species. We found that native plant species interacted, on average, with 124% more microherbivore species than non‐native species among non‐woody plants, and with 175% more among woody plants. Among non‐native plants, microherbivore interactions were best explained by introduced range size, followed by time since introduction, geographic proximity to Europe, and lastly (but not significantly) relatedness. Non‐native plants achieved native levels of microherbivory richness once they reached a range size comparable to native species (≈1 million km^2^ for non‐woody or 270,000 km^2^ for woody plants) or after residing for at least 306 years (non‐woody) or 180 years (woody) since introduction. These results are unexpected, as non‐native plants are often thought to remain trophically isolated due to their lack of co‐evolutionary history (Hill and Kotanen 2009; Pearse and Hipp 2009). Our findings suggest that substantial numbers of microherbivore associations can accumulate within a few centuries, indicating that non‐natives are not necessarily excluded from trophic webs in the long term.

Given that co‐evolution is typically invoked to explain why non‐native plants support fewer trophic interactions than natives, it is striking that relatedness was not a strong predictor in our model. While several studies show that generalist and even specialist herbivores can switch to novel hosts (Castells et al. 2014; García‐Robledo and Horvitz 2011; Pearse and Altermatt 2013), others highlight significant relatedness effects in particular regions (Pearse and Hipp 2009), suggesting scale dependence. Our continental‐scale analysis likely diluted such effects, as non‐natives were classified as congeneric even though they may overlap with congeners only in limited parts of their introduced range. Relatedness may matter regionally, where co‐occurrence with congeners facilitates herbivore spillover, but it is not a dominant predictor at a continental scale. Instead, native‐range proximity explained more variation. Species from nearby regions are more likely to resemble the native flora in terms of traits, climate adaptations and community context. Moreover, microherbivores with broad Eurasian distributions may already have encountered these plants in their native ranges (Cripps et al. 2006). Thus, while relatedness may be more important at smaller spatial scales, our results point to a significant role of ecological similarity and pre‐existing interactions in shaping microherbivory richness at a continental scale.

Introduced range size emerged as the strongest predictor of trophic integration, prompting the question of what drives this relationship (*cf*. Brändle and Brandl 2001). To minimize confounding, we modelled all predictors jointly. For example, time since introduction was positively correlated with range size, suggesting that if analyzed alone, the effect of range size might partly reflect residence time. Yet both variables remained significant when modelled together. Another possible confounder is sampling effort. Plants with larger ranges are more likely to be studied, which may inflate their recorded interactions. Certain plant traits may likewise promote both wide distributions and high microherbivore richness. Nitrogen‐demanding species, for instance, tend to have larger ranges (Segar et al. 2022; Staude et al. 2022) and often invest less in leaf defenses, potentially making them more accessible to microherbivores (Ebeling et al. 2022). Yet in supplementary analyses restricted to the German flora, where sampling effort is relatively high and more homogeneous, the strong range‐size effect persisted even after controlling for nutrient indicator values and leaf nitrogen content (Figure S8; Tables S2 and S3). Thus, the effect is unlikely to be a mere artefact of uneven sampling or correlated traits. Instead, range size likely functions as a proxy for encounter probability (Strong et al. 1984): plants with larger introduced ranges accumulate more trophic interactions simply by being more widespread and frequently encountered.

A corollary of this finding concerns the enemy release hypothesis, which posits that release from enemies facilitates the invasive spread of non‐native plants (Keane and Crawley 2002; Mitchell and Power 2003). If this were the case, widespread non‐natives should host fewer microherbivores; but we found the opposite. This apparent contradiction may have several explanations. First, non‐native species with large ranges across Europe are not necessarily invasive. Many invasive species are locally dominant but geographically restricted, as shown in the British Countryside Survey (Thomas and Palmer 2015a, 2015b). Second, enemy release may be a transient phenomenon: non‐native plants might initially benefit from reduced herbivory, enabling spread, but accumulate herbivores over time as they become more common, consistent with the host naïveté hypothesis (Woodard et al. 2012). This mirrors findings for many invasive species, whose local dominance can decline after a few decades, suggesting that early release effects may fade (Speek et al. 2011, 2015). Such dynamics could produce a positive association between range size and microherbivore richness, even if reduced enemy pressure played a role early in the process of range expansion. That said, our study cannot directly test the enemy release hypothesis. We did not focus on invasive species and our continental‐scale data does not capture local patterns of dominance and interaction.

To explore how native and non‐native plants differ in trophic network structure, we compared the degree of specialisation among their associated microherbivores. Although a large introduced range enabled non‐native plants to support numbers of microherbivore species comparable to natives, the composition of their interactions remained distinct. Non‐native plants were more often associated with generalist microherbivores, which exhibited, on average, twice the host breadth of those interacting with native plants. This indicates that greater spatial and temporal exposure can raise richness (the number of microherbivore species recorded) on non‐native plants, but this speaks to richness only. Other dimensions—microherbivore identities, rates of herbivory, and effects on plants—may tell a different story. Specifically, our results support the idea that microherbivore specialisation is a slow evolutionary process shaped by long‐term co‐evolutionary history, something non‐native species have not yet experienced in their new ranges (Brändle et al. 2008; Kirichenko et al. 2013; Schulz et al. 2025). Native plants then support a unique trophic function, as they sustain a major share of highly specialised microherbivore fauna, including ≈9000 species that rely on just a single native host plant (Figure S4). Non‐native plants may broaden the diet of generalists, but they do not substitute the ecological role of natives in maintaining specialist diversity.

How do our results fit into the broader context of biotic interactions? Much of the debate around non‐native plants in ecological networks has centered on pollination, especially amid concerns about pollinator declines (Hallmann et al. 2017; Salisbury et al. 2015). Early concerns that non‐natives attract fewer pollinators and disrupt native plant reproduction (Morales and Traveset 2009) have been tempered by meta‐analyses showing mixed effects. Many non‐native species are attractive to pollinators, and overall impacts on native pollination success appear limited (Bartomeus et al. 2008; Charlebois and Sargent 2017). Our findings suggest that, like pollination, microherbivore interactions with non‐native plants are more nuanced than previously assumed. While non‐native plants are often assumed to disrupt trophic networks—by outcompeting natives, escaping enemies, or introducing novel pathogens—we find that they can join relatively quickly, albeit skewed toward generalist microherbivores. Meanwhile, highly specialised microherbivores tend to rely on widespread native host plants, a pattern known as network nestedness (Bascompte et al. 2003; Bassi and Staude 2024), suggesting resilience in these interactions and a limited risk of displacement through non‐native plants. In this light, non‐native plants in trophic webs may signal not only disruption, but also the adaptive capacity of ecological networks to absorb novelty.

The generally higher microherbivore interaction richness of native plants, along with the greater prevalence of highly specialised microherbivores they support, underscores the essential role of native flora in sustaining native food webs. Yet our finding that non‐native species which have been present longer and become more widespread in Europe now support microherbivory richness levels comparable to the average native plant challenges the assumption that a lack of co‐evolution renders non‐natives perpetual outsiders. Instead, our results suggest that ecosystems are dynamic and resilient. Newly arriving plant species that become common are not ignored indefinitely; especially generalist microherbivores begin using them. As novel biotic communities emerge, just as they have throughout ecological history (Ordonez et al. 2024; Williams and Jackson 2007), organisms do not passively coexist but actively respond to one another. Non‐native plants need not take millennia to join trophic webs, but may do so over relatively short timescales.

## Author Contributions

I.R.S. conceived the project and supervised L.J.S. and M.W. L.S. and I.R.S. led the main analysis with contributions from M.W. L.J.S., M.W. and I.R.S. jointly wrote the manuscript.

## Peer Review

The peer review history for this article is available at https://www.webofscience.com/api/gateway/wos/peer‐review/10.1111/ele.70247.

## Supporting information


**Figure S1:** Feeding guilds of plant‐feeding organisms in the dataset.
**Figure S2:** Definition of Europe according to the World Checklist of Vascular Plants.
**Figure S3:** Relationship between introduction date and non‐native plant species' area of occupancy (range size) in Europe.
**Figure S4:** Centroids of native ranges for 3533 non‐native plant species.
**Figure S5:** Histogram showing the number of native plant species used by each microherbivore species.
**Figure S6:** Non‐native plants with large European ranges support similar or even higher numbers of microherbivore species compared to native plants.
**Figure S7:** Residence time and specialisation composition of microherbivores on non‐native plants.
**Figure S8:** The effect of plant range size persists after accounting for habitat affinities and traits, and remains robust across the well‐sampled German flora.
**Table S1:** Results from a robust linear mixed‐effects model assessing the effects of plant origin (native vs. non‐native), woodiness (herbaceous vs. woody), and their interaction on the number of associated microherbivore species per plant (log_10_‐transformed).
**Table S2:** Results from a linear mixed‐effects model predicting the number of associated microherbivore species per plant species, using plant range size (log_10_ AOO) and ecological indicator value for nitrogen (EIV N) as fixed effects.
**Table S3:** Results from a linear mixed‐effects model predicting the number of associated microherbivore species per plant species, using plant range size (log_10_ AOO) and leaf nitrogen content per unit dry mass (N per LDM) as fixed effects.

## Data Availability

The data and code that support the findings of this study are openly available on GitHub (https://github.com/istaude/nonnatives‐microherbivores) and archived on Zenodo (https://doi.org/10.5281/zenodo.17214644).
