# Supplementary material for: Non‐Native Plants Attain Native Levels of Microherbivory Richness With Time and Range Expansion
Source: Ecol Lett. 2025 Nov 5;28(11):e70247. doi: 10.1111/ele.70247 (PMC12589859; doi:10.1111/ele.70247)
Supplement: Supplementary file 1 — Figure S1: Feeding guilds of plant‐feeding organisms in the dataset. Figure S2: Definition of Europe according to the World Checklist of Vascular Plants. Figure S3: Relationship between introduction date and non‐native plant species' area of occupancy (range size) in Europe. Figure S4: Centroids of native ranges for 3533 non‐native plant species. Figure S5: Histogram showing the number of native plant species used by each microherbivore species. Figure S6: Non‐native plants with large European ranges support similar or even higher numbers of microherbivore species compared to native plants. Figure S7: Residence time and specialisation composition of microherbivores on non‐native plants. Figure S8: The effect of plant range size persists after accounting for habitat affinities and traits, and remains robust across the well‐sampled German flora. Table S1: Results from a robust linear mixed‐effects model assessing the effects of plant origin (native vs. non‐native), woodiness (herbaceous vs. woody), and their interaction on the number of associated microherbivore species per plant (log10‐transformed). Table S2: Results from a linear mixed‐effects model predicting the number of associated microherbivore species per plant species, using plant range size (log10 AOO) and ecological indicator value for nitrogen (EIV N) as fixed effects. Table S3: Results from a linear mixed‐effects model predicting the number of associated microherbivore species per plant species, using plant range size (log10 AOO) and leaf nitrogen content per unit dry mass (N per LDM) as fixed effects. [file ELE-28-0-s001.docx]

**Supplementary Material**

**Non-native plants attain native levels of microherbivory richness with time and range expansion**

**Authors:** *Lara J. Schulte, Miriam Wahl & Ingmar R. Staude*

**Corresponding author:** Ingmar R. Staude ([ingmar.staude@uni-leipzig.de](mailto:ingmar.staude@uni-leipzig.de))

**Content:**

**Supplementary Figures:**

Fig. S1: Feeding guilds of plant-feeding organisms in the dataset.

Fig. S2: Definition of Europe according to the World Checklist of Vascular Plants

Fig. S3: Relationship between introduction date and non-native plant species’ area of occupancy (range size) in Europe.

Fig. S4: Centroids of native ranges for 3,533 non-native plant species.

Fig. S5: Histogram showing the number of native plant species used by each microherbivore species.

Fig. S6: Non-native plants with large European ranges support similar or even higher numbers of microherbivore species compared to native plants.

Fig. S7: Residence time and specialization composition of microherbivores on non-native plants.

Fig. S8: The effect of plant range size persists after accounting for habitat affinities and traits, and remains robust across the well-sampled German flora.

**Supplementary Tables:**

Table S1: Results from a robust linear mixed-effects model assessing the effects of plant origin (native vs. non-native), woodiness (herbaceous vs. woody), and their interaction on the number of associated microherbivore species per plant (log₁₀-transformed).

Table S2: Results from a linear mixed-effects model predicting the number of associated microherbivore species per plant species, using plant range size (log₁₀ AOO) and ecological indicator value for nitrogen (EIV N) as fixed effects.

Table S3: Results from a linear mixed-effects model predicting the number of associated microherbivore species per plant species, using plant range size (log₁₀ AOO) and leaf nitrogen content per unit dry mass (N per LDM) as fixed effects.

**References**


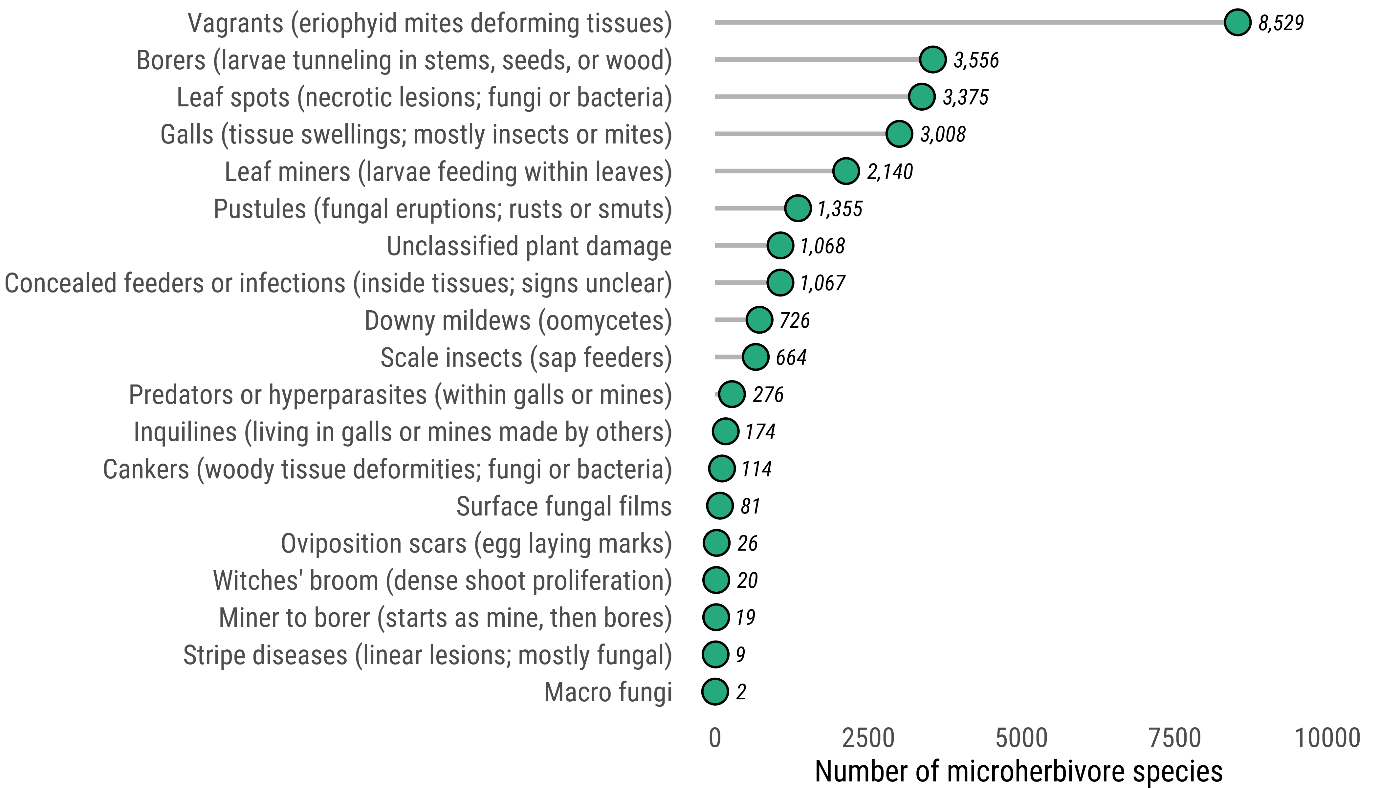


**Fig. S1: Feeding guilds of plant-feeding organisms in the dataset.** Lollipop plot showing the number of microherbivore species per feeding guild (point labels give counts). Records with missing guild annotation are not shown (NA = 184).


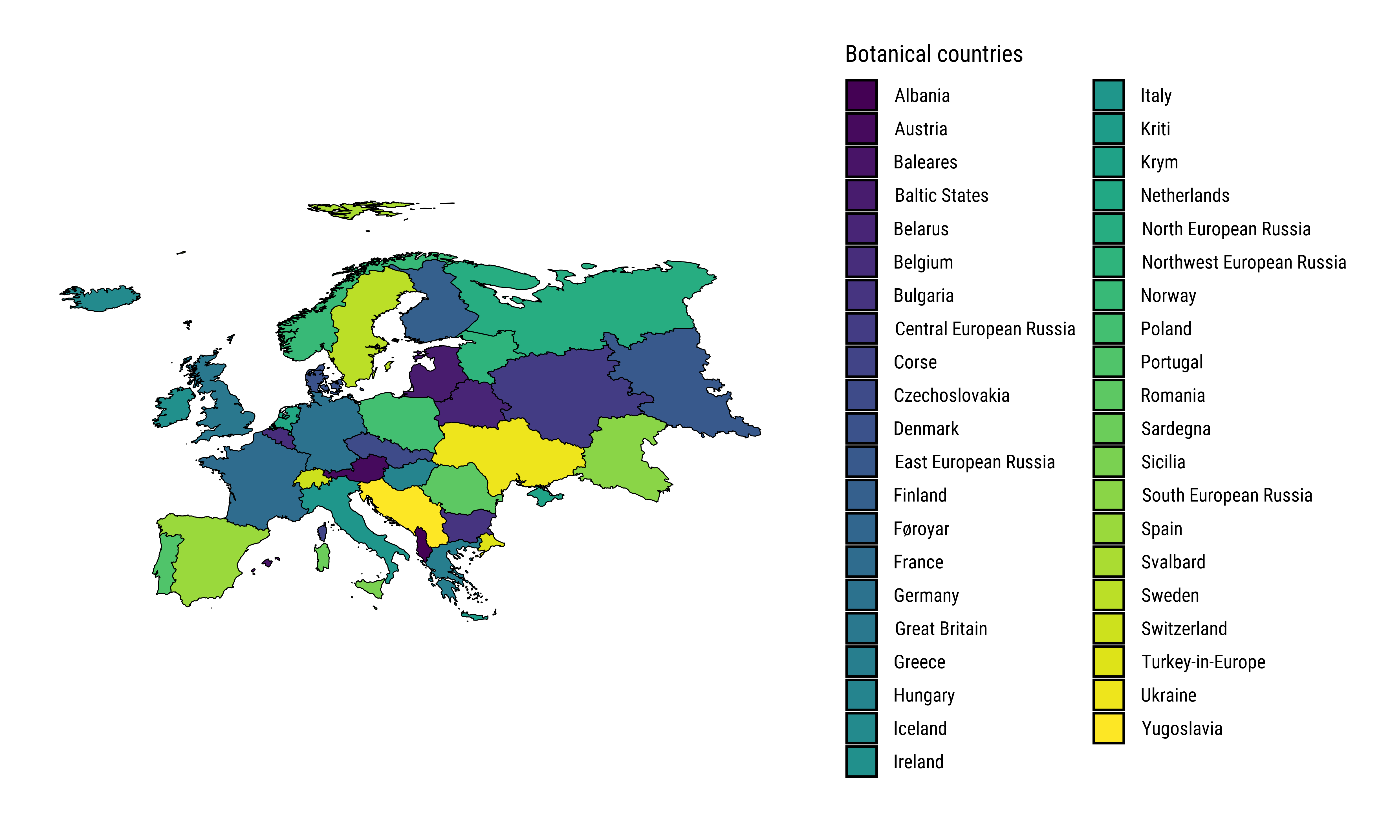


**Fig. S2: Definition of Europe according to the World Checklist of Vascular Plants.** Shown are botanical countries (TDWG Level 3 regions).


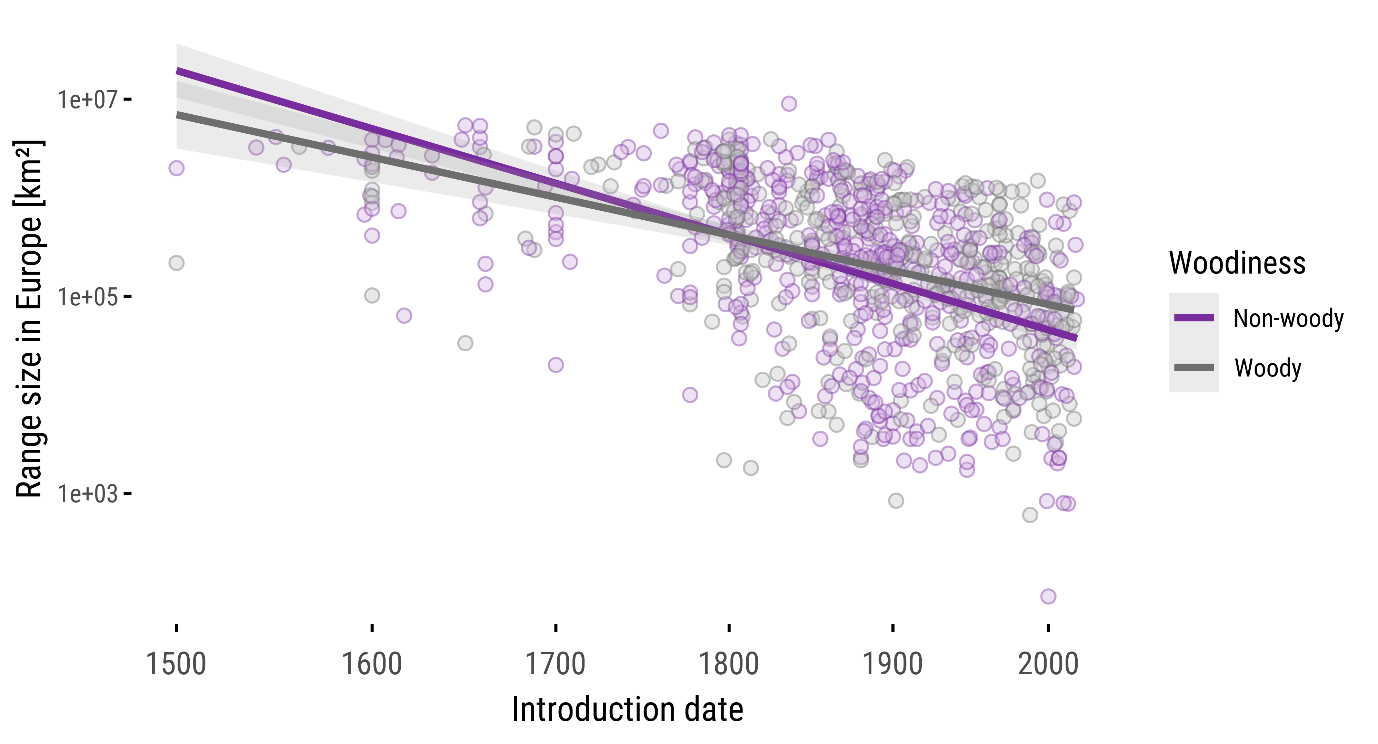


**Fig. S3: Relationship between introduction date and non-native plant species’ area of occupancy (range size) in Europe.** Semi-transparent points show observed values (log_10_ scale on y-axis) for each non-native plant species (n=942); lines and shaded bands represent predicted means with 95% confidence intervals. Non-woody species are shown in purple, woody species in grey. The relationships are significant for both non-woody (β = –0.00512, 95% CI: –0.00578 to –0.00445) and woody species (β = –0.00369, 95% CI: –0.00450 to –0.00288).


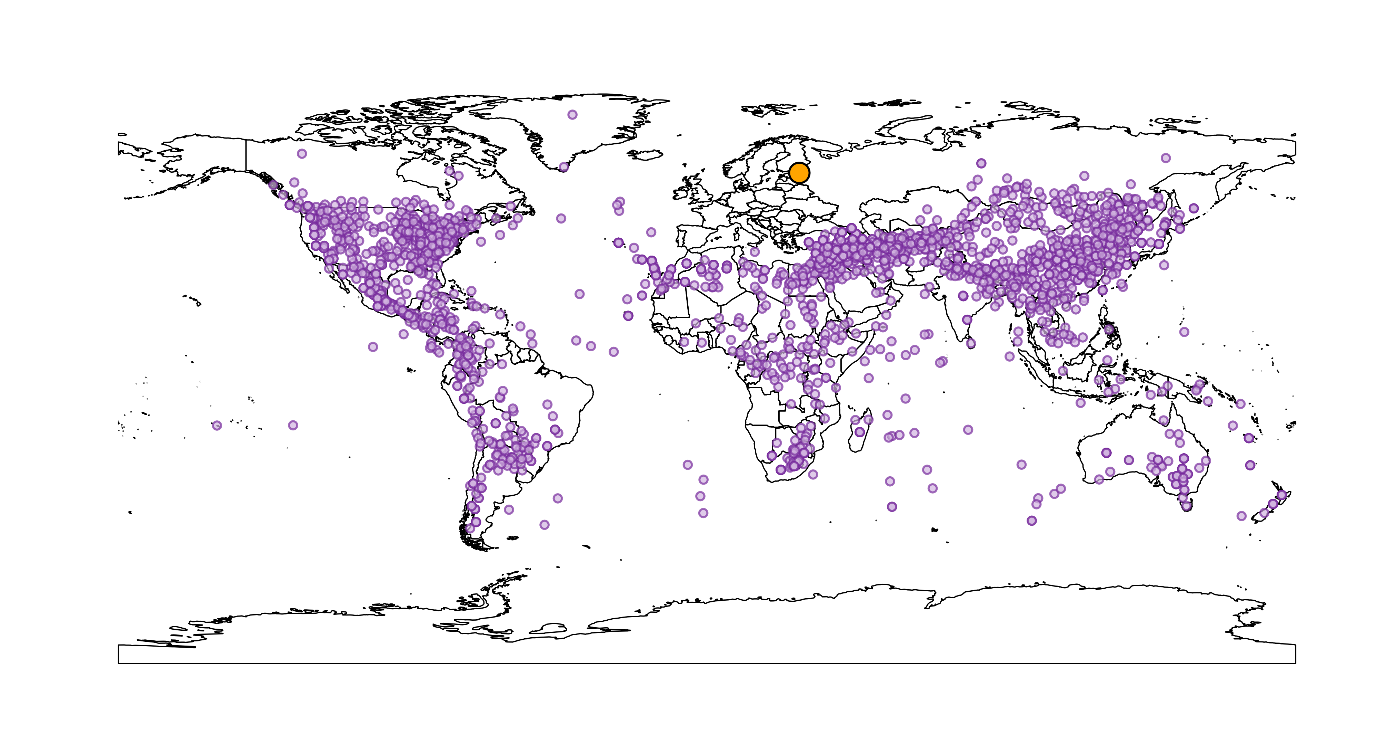


**Fig. S4: Centroids of native ranges for 3,533 non-native plant species.** Purple dots indicate individual species' centroids; the orange dot shows the geographic centre of Europe (see Fig. S2 and Methods).


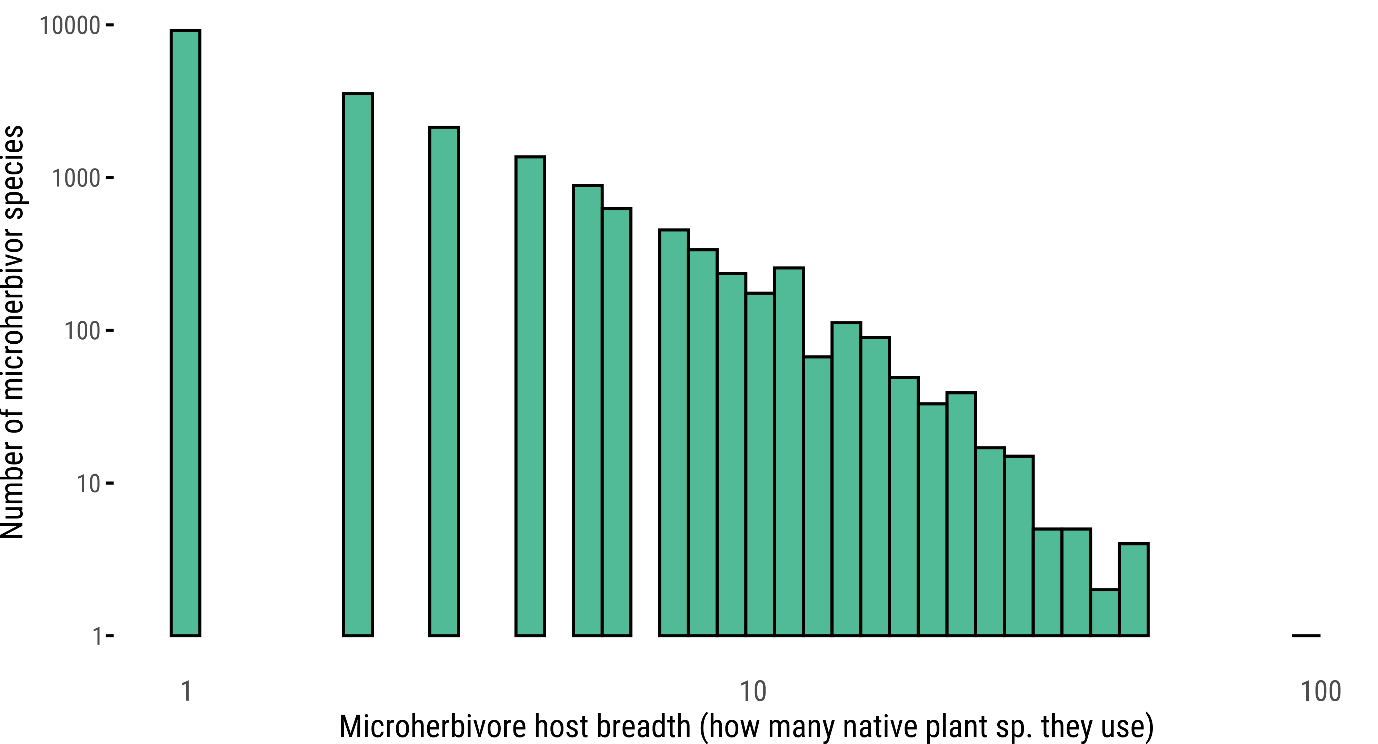


**Fig. S5: Histogram showing the number of native plant species used by each microherbivore species.** Displayed are microherbivores that exclusively interact with native plant species, i.e., have no non-native plant species in their interaction portfolio. Among these microherbivores, a substantial proportion (47%, or 9,216 of 19,667species) is associated with just one native plant species. Both axes are on a log_10_-scale.


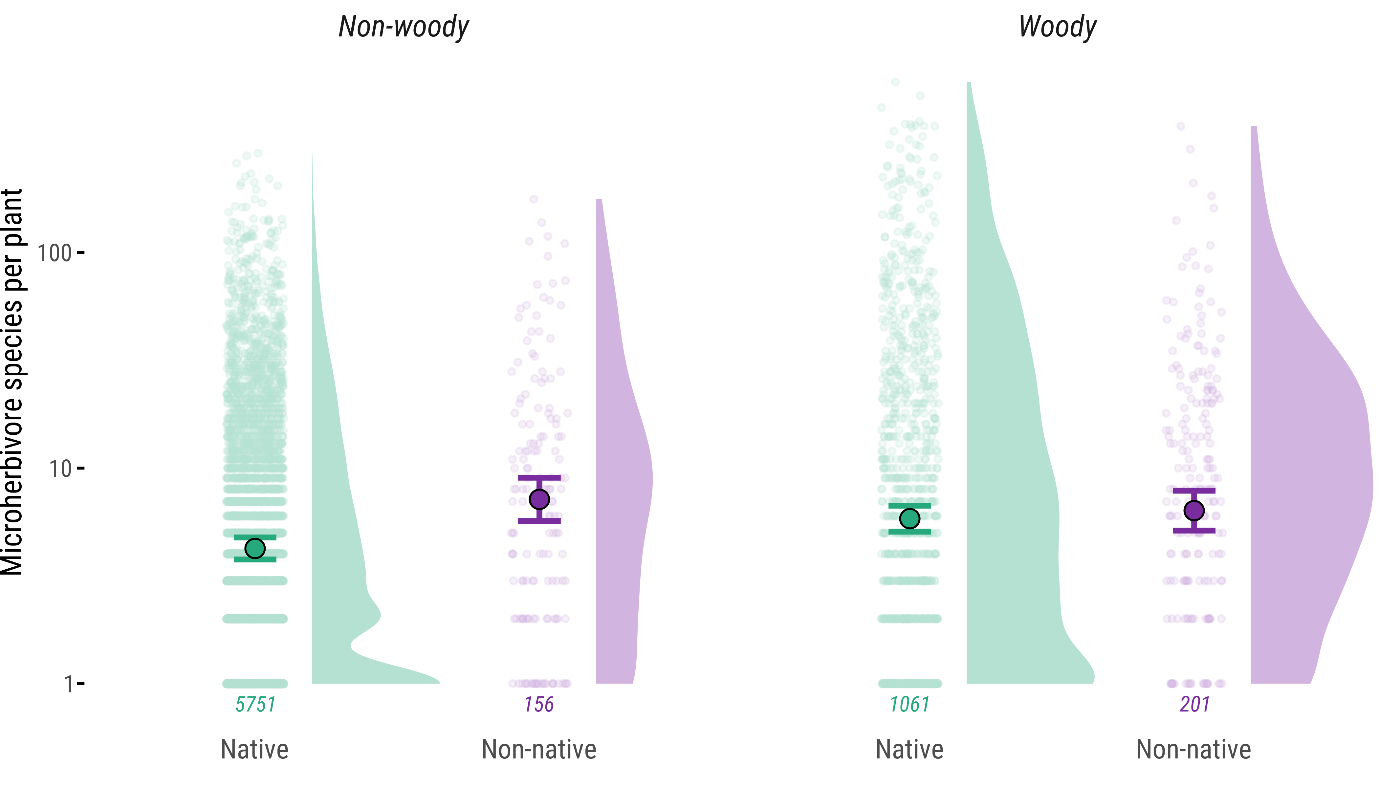


**Fig. S6: Non-native plants with large European ranges support similar or even higher numbers of microherbivore species compared to native plants.** Displayed are non-native plants with large European range sizes (≈1.1 million km² for non-woody and ≈270,000 km² for woody species), which were previously shown to best predict native-like levels of trophic integration (Fig. 2). Plants are grouped by woodiness (non-woody vs. woody) and by origin (native = green, non-native = purple). Each point represents one plant species (semi-transparent), with coloured points indicating group means and error bars showing 95% confidence intervals. Density plots show the distribution of values. The y-axis is displayed on a log₁₀ scale. Sample sizes are indicated below the point clouds.


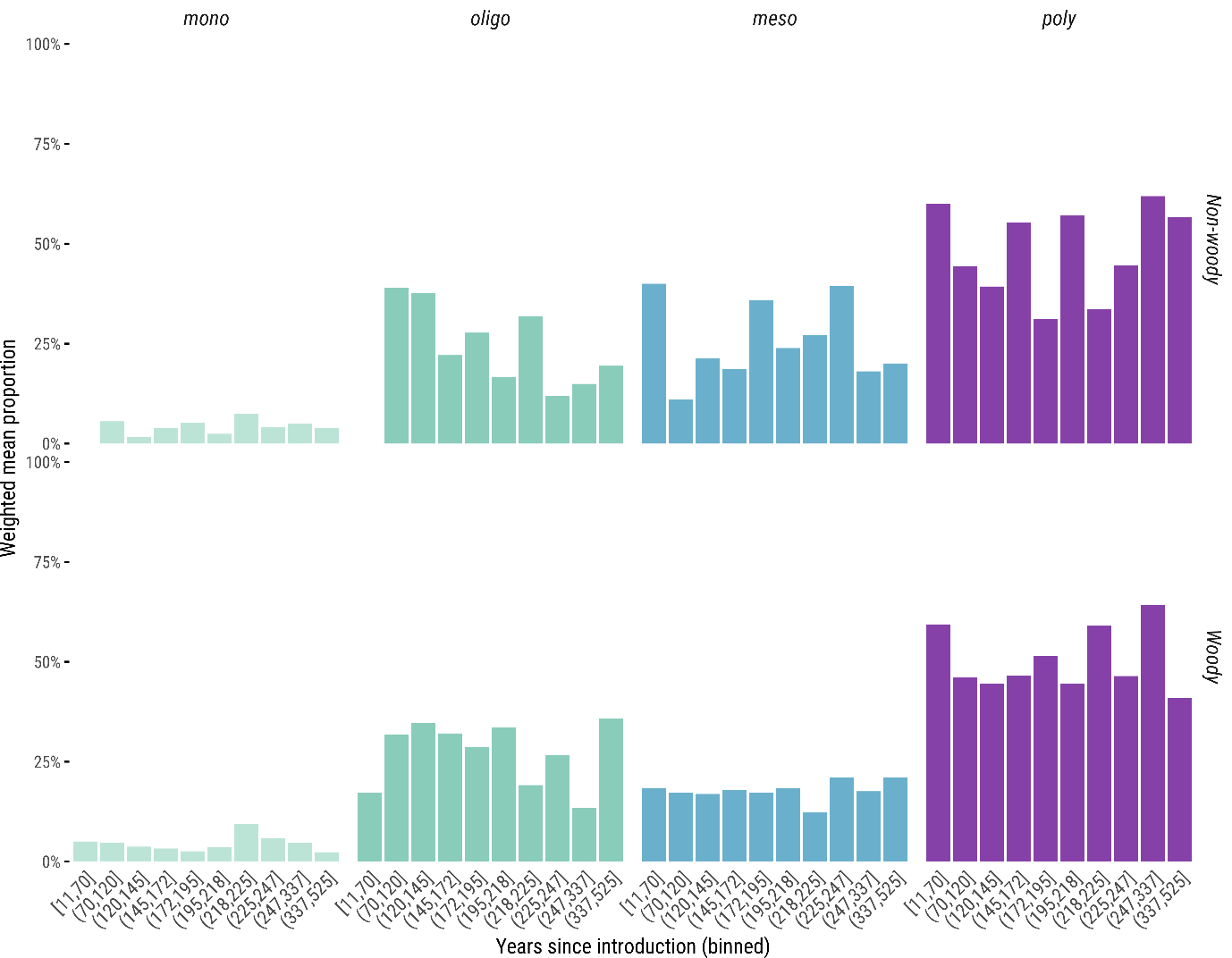


**Fig. S7: Residence time and specialization composition of microherbivores on non-native plants.** For trophically well-integrated non-native species with known introduction dates (n = 307), years since introduction were binned. Within each bin we computed the weighted mean per-plant proportion of microherbivores in each specialization class (mono/oligo/meso/poly), weighting by each plant’s total recorded interactions. Rows show woody vs. non-woody plants; columns show specialization classes.


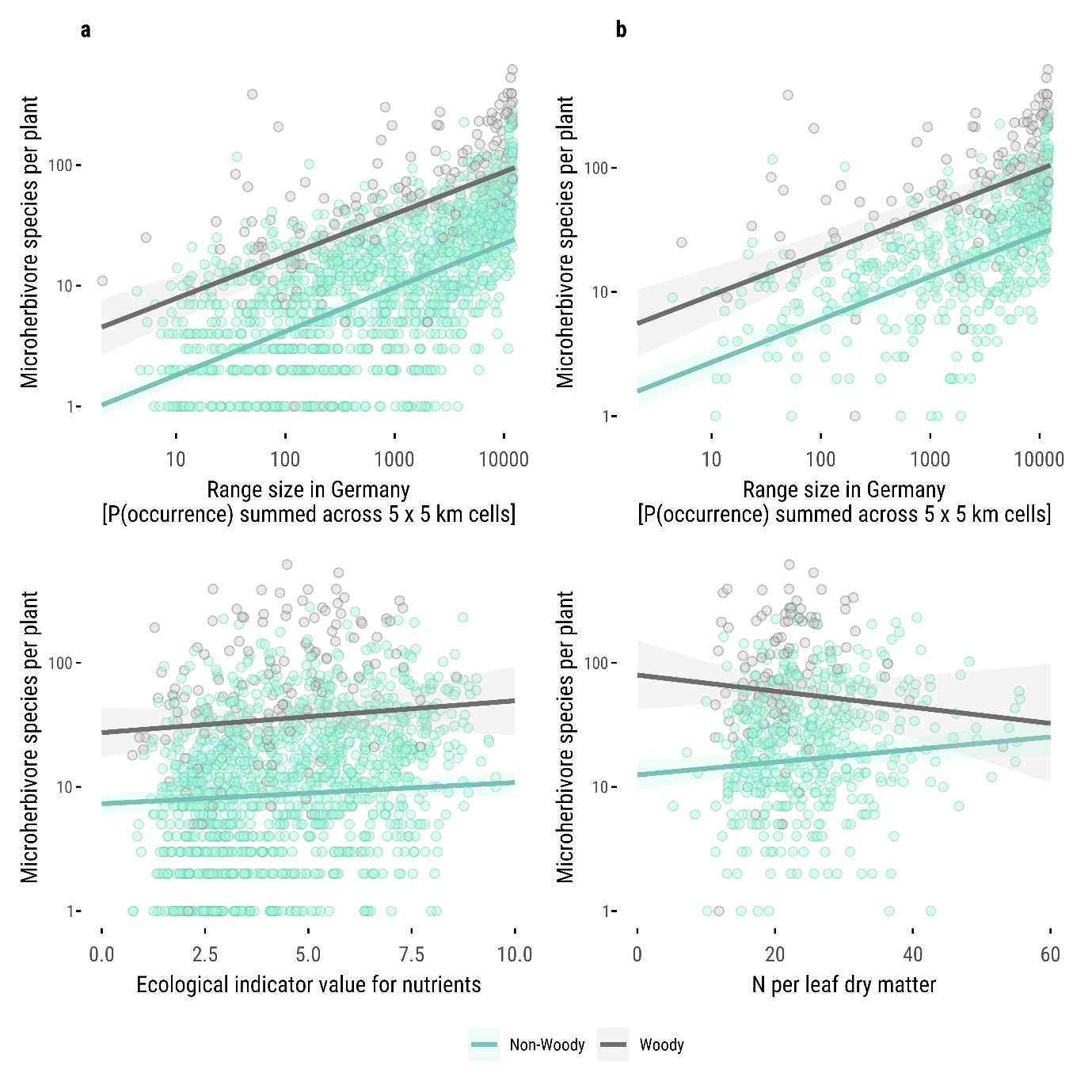
**Fig. S8: The effect of plant range size persists after accounting for habitat affinities and traits, and remains robust across the well-sampled German flora.** (a) Model including both range size and the ecological indicator value for nutrients as predictors of microherbivory richness. (b) Model including both range size and leaf nitrogen (N) content per unit dry mass. In both models, plant family was included as a random effect. Each point represents a plant species from the German flora, a subset chosen because sampling effort is comparatively high and homogeneous. Range size estimates for each plant species in Germany were taken from (Eichenberg *et al.* 2021) and expressed as the summed occurrence probability across 5 × 5 km² grid cells. Ecological indicator values were obtained from the EIVE database (Dengler *et al.* 2023), and leaf nitrogen content from the TRY database (Kattge *et al.* 2020). All datasets were taxonomically harmonized using the World Checklist of Vascular Plants. Model results are provided in Tables S2 and S3.

**Supplementary Tables**

**Table S1: Results from a robust linear mixed-effects model assessing the effects of plant origin (native vs. non-native), woodiness (herbaceous vs. woody), and their interaction on the number of associated microherbivore species per plant (log₁₀-transformed).** Plant family was included as a random effect to account for phylogenetic non-independence.

|  | **log10(number of microherbivores)** | | |  |
| --- | --- | --- | --- | --- |
| *Predictors* | *Estimates* | *CI* | *p* |  |
| (Intercept) | 0.55 | 0.52 – 0.58 | **<0.001** |  |
| plant origin [Non-native] | -0.32 | -0.35 – -0.30 | **<0.001** |  |
| woodiness [Woody] | 0.18 | 0.14 – 0.22 | **<0.001** |  |
| plant origin [Non-native] x woodiness [Woody] | -0.08 | -0.13 – -0.03 | **0.002** |  |
| **Random Effects** | | | |  |
| σ^2^ | 0.25 | | |  |
| τ_00_ _family_ | 0.01 | | |  |
| ICC | 0.06 | | |  |
| N _family_ | 193 | | |  |
| Observations | 10265 | | |  |
| Marginal R^2^ / Conditional R^2^ | 0.086 / 0.137 | | |  |

**Table S2: Results from a linear mixed-effects model predicting the number of associated microherbivore species per plant species, using plant range size (log₁₀ AOO) and ecological indicator value for nitrogen (EIV N) as fixed effects.** Woodiness and all interactions were included, and plant family was included as a random effect to account for phylogenetic non-independence. Model fit and variance components are reported at the bottom of the table.

|  | **log10(number of microherbivores)** | | |
| --- | --- | --- | --- |
| *Predictors* | *Estimates* | *CI* | *p* |
| (Intercept) | -0.18 | -0.28 – -0.09 | **<0.001** |
| log10(AOO) | 0.36 | 0.34 – 0.39 | **<0.001** |
| Woodiness [Woody] | 0.61 | 0.35 – 0.87 | **<0.001** |
| EIV N | 0.02 | 0.01 – 0.03 | **0.004** |
| log10(AOO) × Woodiness [Woody] | -0.01 | -0.09 – 0.06 | 0.731 |
| Woodiness [Woody] × EIV N | 0.01 | -0.04 – 0.05 | 0.706 |
| **Random Effects** | | | |
| σ^2^ | 0.12 | | |
| τ_00_ _family_ | 0.09 | | |
| ICC | 0.43 | | |
| N _family_ | 113 | | |
| Observations | 1462 | | |
| Marginal R^2^ / Conditional R^2^ | 0.422 / 0.671 | | |

**Table S3: Results from a linear mixed-effects model predicting the number of associated microherbivore species per plant species, using plant range size (log₁₀ AOO) and leaf nitrogen content per unit dry mass (N per LDM) as fixed effects.** Woodiness and all interactions were included, and plant family was included as a random effect to account for phylogenetic non-independence. Model fit and variance components are reported at the bottom of the table.

|  | **log10(number of microherbivores)** | | |
| --- | --- | --- | --- |
| *Predictors* | *Estimates* | *CI* | *p* |
| (Intercept) | -0.03 | -0.20 – 0.13 | 0.673 |
| log10(AOO) | 0.34 | 0.31 – 0.38 | **<0.001** |
| Woodiness [Woody] | 0.83 | 0.49 – 1.17 | **<0.001** |
| N per LDM | 0.01 | 0.00 – 0.01 | **0.006** |
| log10(AOO) × Woodiness [Woody] | -0.01 | -0.09 – 0.08 | 0.865 |
| Woodiness [Woody] × N per LDM | -0.01 | -0.02 – 0.00 | 0.064 |
| **Random Effects** | | | |
| σ^2^ | 0.08 | | |
| τ_00_ _family_ | 0.10 | | |
| ICC | 0.55 | | |
| N _family_ | 90 | | |
| Observations | 617 | | |
| Marginal R^2^ / Conditional R^2^ | 0.390 / 0.726 | | |

**References**

Dengler, J., Jansen, F., Chusova, O., Hüllbusch, E., Nobis, M.P., Van Meerbeek, K., *et al.* (2023). Ecological Indicator Values for Europe (EIVE) 1.0. *Vegetation Classification and Survey*, 4, 7–29.

Eichenberg, D., Bowler, D.E., Bonn, A., Bruelheide, H., Grescho, V., Harter, D., *et al.* (2021). Widespread decline in Central European plant diversity across six decades. *Global Change Biology*, 27, 1097–1110.

Kattge, J., Bönisch, G., Díaz, S., Lavorel, S., Prentice, I.C., Leadley, P., *et al.* (2020). TRY plant trait database–enhanced coverage and open access. *Global change biology*, 26, 119–188.
